# Beyond Breslow Thickness: Reproducibility and Histopathologic Concordance of Ultra-High-Frequency Ultrasound in Primary Cutaneous Melanoma

**DOI:** 10.3390/diagnostics16203416

**Published:** 2026-10-20

**Authors:** Anna Russo, Vittorio Patanè, Annalisa Di Maio, Lucrezia Bucciero, Renato Franco, Teresa Troiani, Giuseppe Argenziano, Alfonso Reginelli

**Affiliations:** 1 Precision Medicine Department, University of Campania “Luigi Vanvitelli”, Piazza Luigi Miraglia 2, 80138 Naples, Italy; 2 Pathology Division, Department of Mental and Physical Health and Preventive Medicine, University of Campania “Luigi Vanvitelli”, 80138 Naples, Italy; 3 Dermatology Unit, Department of Mental and Physical Health and Preventive Medicine, University of Campania “Luigi Vanvitelli”, 80138 Naples, Italy; giuseppe.argenziano@unicampania.it

**Keywords:** melanoma, ultra-high-frequency ultrasound, UHFUS, Breslow thickness, reproducibility, agreement, histopathology

## Abstract

**Background:** Ultra-high-frequency ultrasound (UHFUS) provides high-resolution assessment of superficial skin lesions, but evidence in melanoma has largely focused on preoperative thickness estimation. We evaluated the reproducibility of a structured UHFUS assessment and its histopathologic concordance in primary cutaneous melanoma. **Methods:** This single-center retrospective study included 320 treatment-naïve, histologically confirmed primary cutaneous melanomas examined with UHFUS (48–70 MHz) before excision. Two experienced radiologists independently assessed tumor thickness, horizontal diameter, surface discontinuity, deepest involved compartment, boundary definition, morphology, echostructure, and separate perilesional hypoechoic foci. Histopathology served as the reference standard. Reproducibility was assessed using ICC and κ statistics; UHFUS–Breslow agreement was evaluated using Lin’s concordance correlation coefficient (CCC) and Bland–Altman analysis. **Results:** Interobserver reproducibility was excellent for tumor thickness (ICC 0.96; 95% CI, 0.95–0.97) and horizontal diameter (ICC 0.94; 95% CI, 0.92–0.95). UHFUS thickness showed excellent concordance with Breslow thickness (CCC 0.95; 95% CI, 0.94–0.96 for both readers), with small positive biases (+0.12 and +0.10 mm). Exact agreement across predefined thickness strata was 90.0%. Surface discontinuity showed high sensitivity and specificity for histopathologic ulceration, whereas perilesional hypoechoic foci were highly specific but moderately sensitive for microsatellitosis. Increased tumor-infiltrating lymphocytes and an associated nevus component independently predicted greater UHFUS overestimation. **Conclusions:** Structured UHFUS provides reproducible quantitative and qualitative preoperative assessment of primary melanoma beyond thickness alone and may serve as a complementary imaging tool within specialized multidisciplinary pathways, while histopathology remains the reference standard for definitive diagnosis and staging.

## 1. Introduction

Cutaneous melanoma is a biologically heterogeneous malignancy in which accurate assessment of the primary lesion remains central to staging and clinical management [1,2,3]. Histopathology is the reference standard for diagnosis and provides multiple parameters relevant to prognostic stratification [4,5]. Breslow thickness and ulceration directly contribute to pathological T classification, while additional features, including histological subtype, growth phase, microsatellitosis, mitotic activity, tumor-infiltrating lymphocytes, regression, lymphovascular invasion, neurotropism, associated nevus component, and margin status, contribute to a comprehensive pathological description of the primary tumor [6,7,8,9,10,11].

Current Italian Association of Medical Oncology (AIOM) recommendations similarly support structured histopathologic reporting of primary melanoma. These variables are, by definition, microscopic findings and should not be assumed to have direct imaging equivalents. Nevertheless, they provide a clinically meaningful framework for investigating which aspects of the primary lesion can be assessed non-invasively before excision.

High- and ultra-high-frequency ultrasound have been increasingly applied to superficial lesions, including dermatologic and dermato-oncologic applications [12,13]. Increasing transducer frequency improves spatial resolution while reducing tissue penetration, making very-high- and ultra-high-frequency ultrasound particularly suitable for superficial tissues. Modern UHFUS systems can provide detailed visualization of the epidermal–dermal region, dermis, dermal–subcutaneous interface, and superficial hypodermis [14,15,16].

The most established application of UHFUS in melanoma is preoperative tumor-thickness assessment. Several studies have demonstrated a close relationship between sonographically measured thickness and histopathologic Breslow thickness, particularly when high-frequency transducers are used [17,18,19,20,21]. However, correlation alone does not establish measurement agreement. Systematic differences may arise because UHFUS and Breslow thickness use different superficial measurement landmarks and because histopathologic processing, tissue composition, and residual tissue compression during scanning may affect the two measurements differently [22].

In addition, limiting UHFUS evaluation to tumor thickness may underestimate the amount of morphological information provided by high-resolution imaging [23]. The relationship of the lesion with the skin layers, definition of its deep and lateral boundaries, surface contour, internal architecture, and vascular pattern can potentially be assessed before excision. Small perilesional hypoechoic foci may also be visible in selected cases.

Particular caution is required when relating UHFUS findings to ulceration and depth of invasion. Histopathologic ulceration is defined microscopically and cannot be reduced to an irregular or interrupted surface on ultrasound [24,25]. For ulceration and depth of invasion, UHFUS provides related but not necessarily equivalent imaging information: surface discontinuity can be compared with histopathologic ulceration, whereas lesion depth can be described according to the deepest involved skin compartment.

We therefore developed a predefined UHFUS assessment of primary cutaneous melanoma based on a limited number of quantitative and morphological variables with plausible anatomical correlates. The primary objectives were to assess interobserver reproducibility of UHFUS tumor thickness and its agreement with histopathologic Breslow thickness. Secondary objectives were to evaluate the reproducibility of selected morphological features, their relationship with relevant histopathologic findings, agreement across clinically relevant thickness categories, and potential histopathologic determinants of UHFUS–histopathology thickness discrepancy.

## 2. Methods

The protocol for the present retrospective analysis was approved by the local Ethics Committee on 1 August 2026 (Protocol No. 2026/15698), after completion of the routine clinical data-acquisition period (January 2025–July 2026). The present investigation was therefore conducted as a retrospective, non-interventional analysis of data and UHFUS examinations previously acquired during routine clinical practice. No additional examinations, procedures, or modifications of patient management were performed for research purposes. The requirement for individual informed consent was waived by the Ethics Committee because of the retrospective nature of the study and the use of data collected during routine clinical care. Patient data were processed in accordance with Regulation (EU) 2016/679 (General Data Protection Regulation, GDPR) and applicable Italian data-protection legislation [26]. Data used for the analysis were pseudonymized before analysis and were accessible only to authorized study personnel. 

### 2.1. Study Design and Population

This single-center retrospective observational study was conducted at the University Hospital “Luigi Vanvitelli”, Naples, Italy. Reporting of the diagnostic-accuracy components of the study was guided by the Standards for Reporting Diagnostic Accuracy Studies (STARD 2015), whereas the reliability and agreement components were reported according to the Guidelines for Reporting Reliability and Agreement Studies (GRRAS) [27,28]. The completed STARD and GRRAS checklists are provided as Supplementary Materials. Consecutive treatment-naïve patients referred by dermatologists for UHFUS assessment of a clinically suspicious melanocytic skin lesion between January 2025 and July 2026 were screened for inclusion. UHFUS was performed before any biopsy, partial excision, or local treatment of the target lesion. In routine clinical practice, UHFUS was requested by the referring dermatologist as part of the preoperative assessment of a clinically suspicious melanocytic lesion. After image acquisition, a radiology report was issued to the referring clinical team before definitive histopathologic assessment. The report included lesion thickness and relevant sonographic characteristics identified during the examination. UHFUS findings were not used as an independent determinant of excision margins, sentinel lymph-node biopsy eligibility, or other therapeutic decisions.

Eligible lesions were required to have technically adequate UHFUS images and subsequent definitive histopathologic assessment. Technical adequacy was defined as complete visualization of the target lesion with sufficiently clear superficial and deep boundaries to permit reliable thickness measurement and anatomical assessment. Examinations were considered technically inadequate when incomplete lesion coverage, acquisition artefacts, or insufficient visualization of the lesion–skin interfaces prevented confident identification of these boundaries. Technical adequacy was assessed during eligibility screening, before the independent blinded image-review phase. Only lesions histologically confirmed as primary cutaneous invasive melanoma were included in the final study cohort. This restriction was predefined because the study was designed to assess the reproducibility of UHFUS-derived quantitative and morphological features and their concordance with histopathologic characteristics in primary melanoma, rather than the diagnostic accuracy of UHFUS for differentiating melanoma from other cutaneous lesions. Accordingly, no binary UHFUS classification as melanoma versus non-melanoma was assigned. Patients were excluded if the target lesion had undergone previous biopsy or treatment, if UHFUS image quality was insufficient for reliable assessment, or if definitive histopathology did not confirm melanoma.

**Figure 1:**
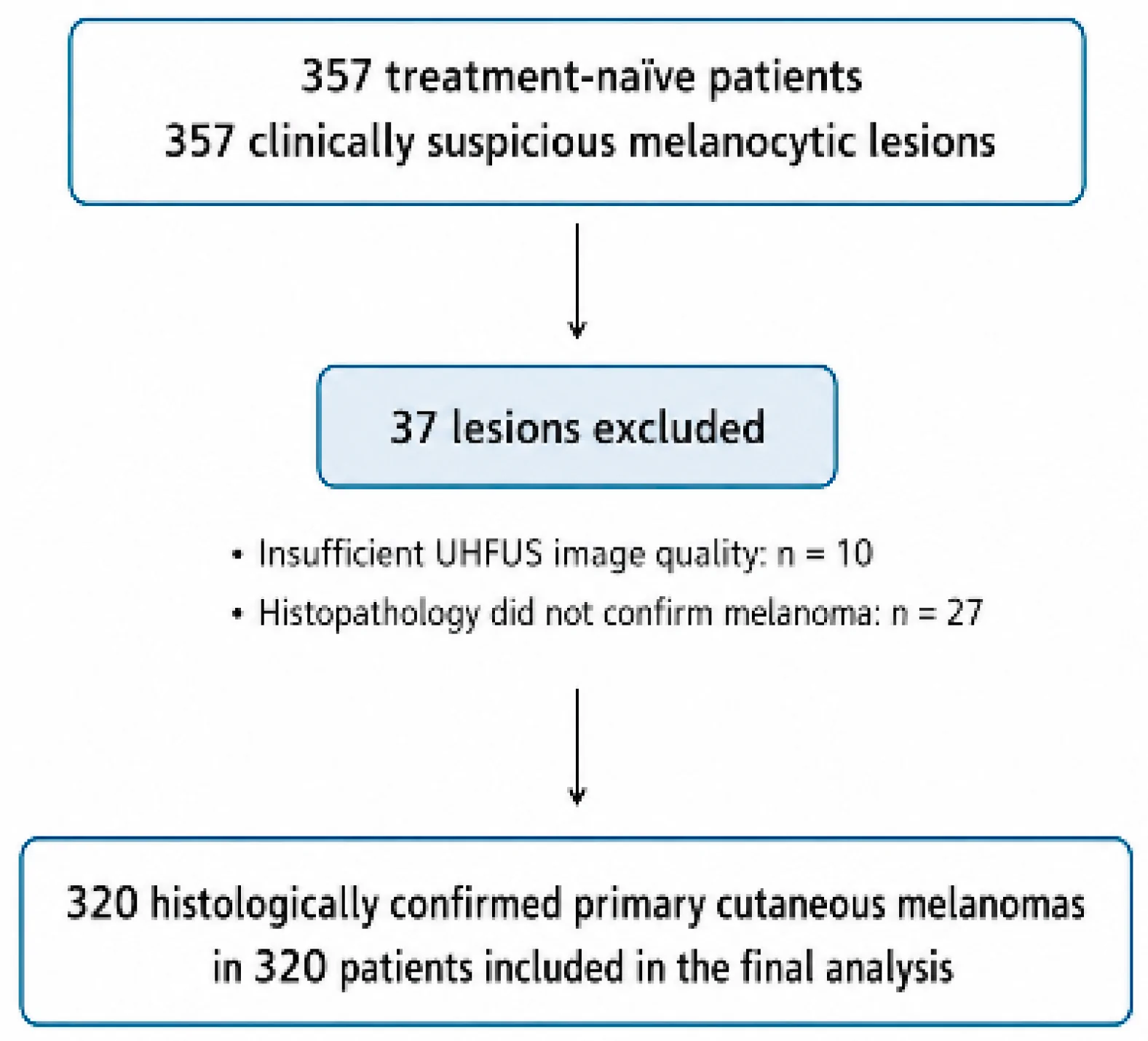
Study flow diagram. Flowchart of patient selection. Consecutive treatment-naïve patients referred for preoperative UHFUS assessment of a clinically suspicious melanocytic lesion were screened. Lesions were excluded because of insufficient UHFUS image quality (*n* = 10) or lack of histopathologic confirmation of melanoma (*n* = 27). The final study population comprised 320 histologically confirmed primary cutaneous melanomas.

During the study period, 357 patients with 357 clinically suspicious melanocytic lesions underwent preoperative UHFUS. Thirty-seven lesions were excluded because of technically inadequate UHFUS examinations (*n* = 10) or absence of histopathologic confirmation of melanoma (*n* = 27). These lesions were not included in the radiologic–histopathologic concordance analyses because histologic confirmation of primary melanoma was a predefined eligibility criterion. Among the 27 lesions excluded because histopathology did not confirm melanoma, final diagnoses included melanocytic nevi (*n* = 15), pigmented basal cell carcinomas (*n* = 5), dermatofibromas (*n* = 2), and other cutaneous lesions (*n* = 5). The final study population comprised 320 histologically confirmed primary cutaneous melanomas in 320 patients (Figure 1).

### 2.2. UHFUS Examination

All examinations were performed in the same facility using a Vevo 3100 system (FUJIFILM VisualSonics, Toronto, Canada) equipped with a 48–70 MHz linear transducer.

This technical setting corresponds to the UHFUS platform and acquisition approach already used in the institution for dedicated skin and soft tissue expert radiology. Scans were acquired in longitudinal and transverse planes using a thin layer of coupling gel, with a stand-off pad when needed to optimize visualization of superficial structures; B-mode imaging was systematically obtained and Color Doppler was added when technically feasible.

All examinations were retrospectively reviewed independently by the same two radiologists who had performed the original UHFUS examinations. During the formal research review, the readers assessed each case separately and had no access to each other’s measurements or classifications. Images were presented in anonymized and randomized order, and definitive histopathologic reports and histopathologic results were not available during image review. Because both readers had participated in the original clinical examinations, however, complete absence of prior familiarity with individual lesions could not be guaranteed. Accordingly, blinding in the present study refers specifically to the information available during the retrospective image-review session rather than to complete clinical naïveté. 

### 2.3. Image Review

For the study analysis, all stored examinations were independently reviewed by both radiologists. This independent assessment concerned image interpretation; image acquisition itself was not independently repeated by both readers. Readers were blinded to definitive histopathologic findings and to each other’s assessment. Cases were reviewed in randomized and anonymized order.

**Figure 2:**
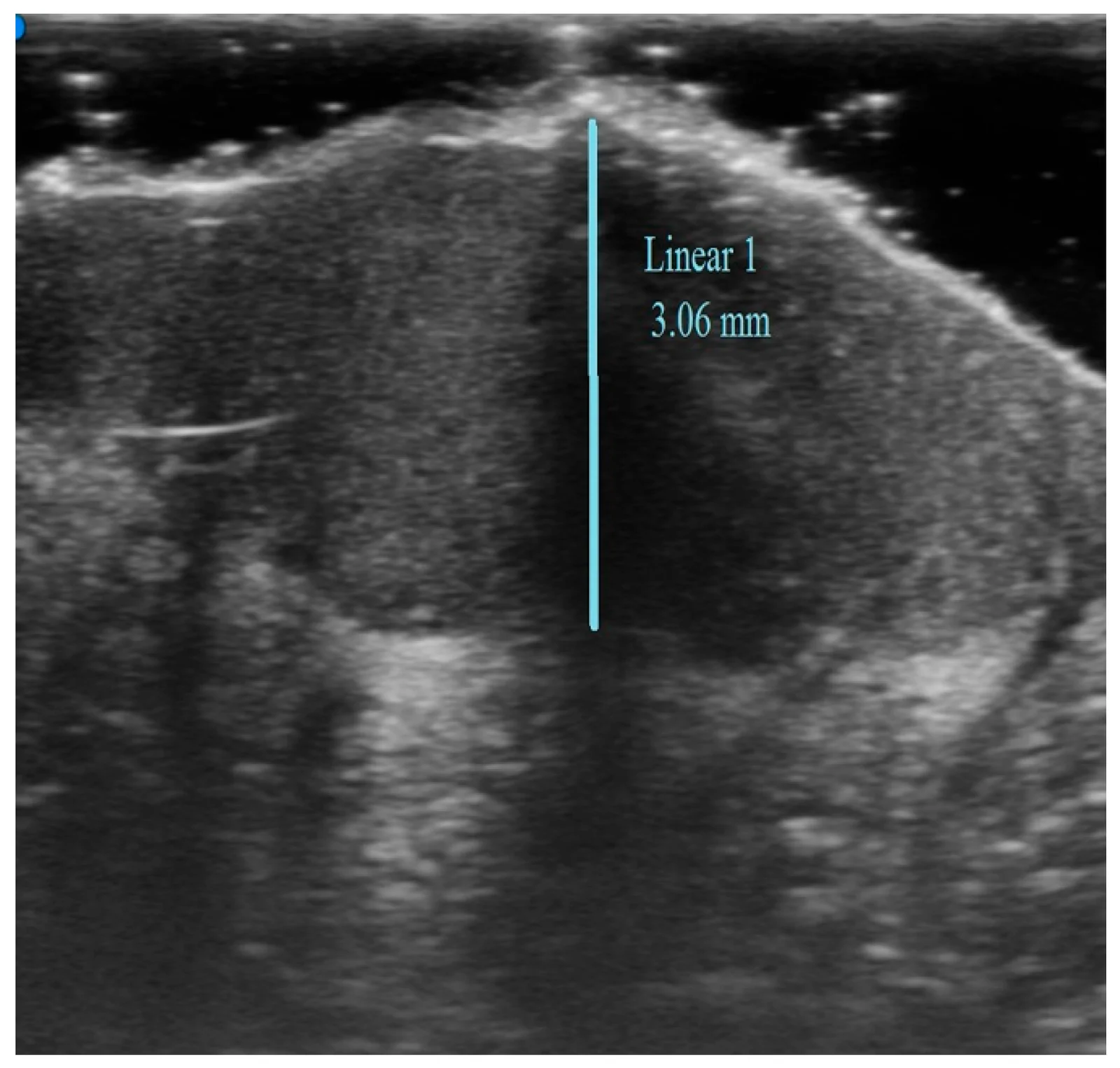
UHFUS assessment of melanoma thickness and comparison with histopathologic Breslow thickness. Representative UHFUS image showing a maximum tumor thickness of 3.06 mm, compared with a histopathologic Breslow thickness of 2.9 mm after excision.

**Figure 3:**
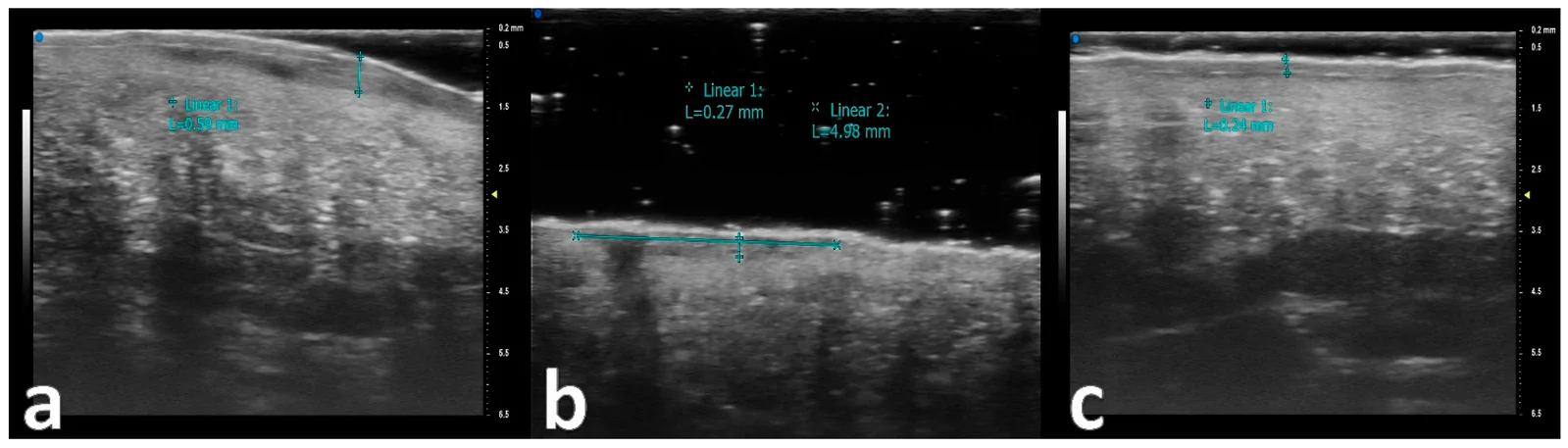
Representative UHFUS appearances of different growth configurations in primary cutaneous melanoma: (**a**) Melanoma showing a relatively greater vertical component, with maximum UHFUS thickness of 0.59 mm. (**b**) Predominantly horizontal growth pattern, characterized by broad lateral extension (4.98 mm) and limited vertical thickness (0.27 mm). (**c**) Thin superficial melanoma with limited vertical extension (0.24 mm). These examples illustrate how UHFUS can depict differences in the balance between horizontal and vertical lesion development. UHFUS, ultra-high-frequency ultrasound.

**Figure 4:**
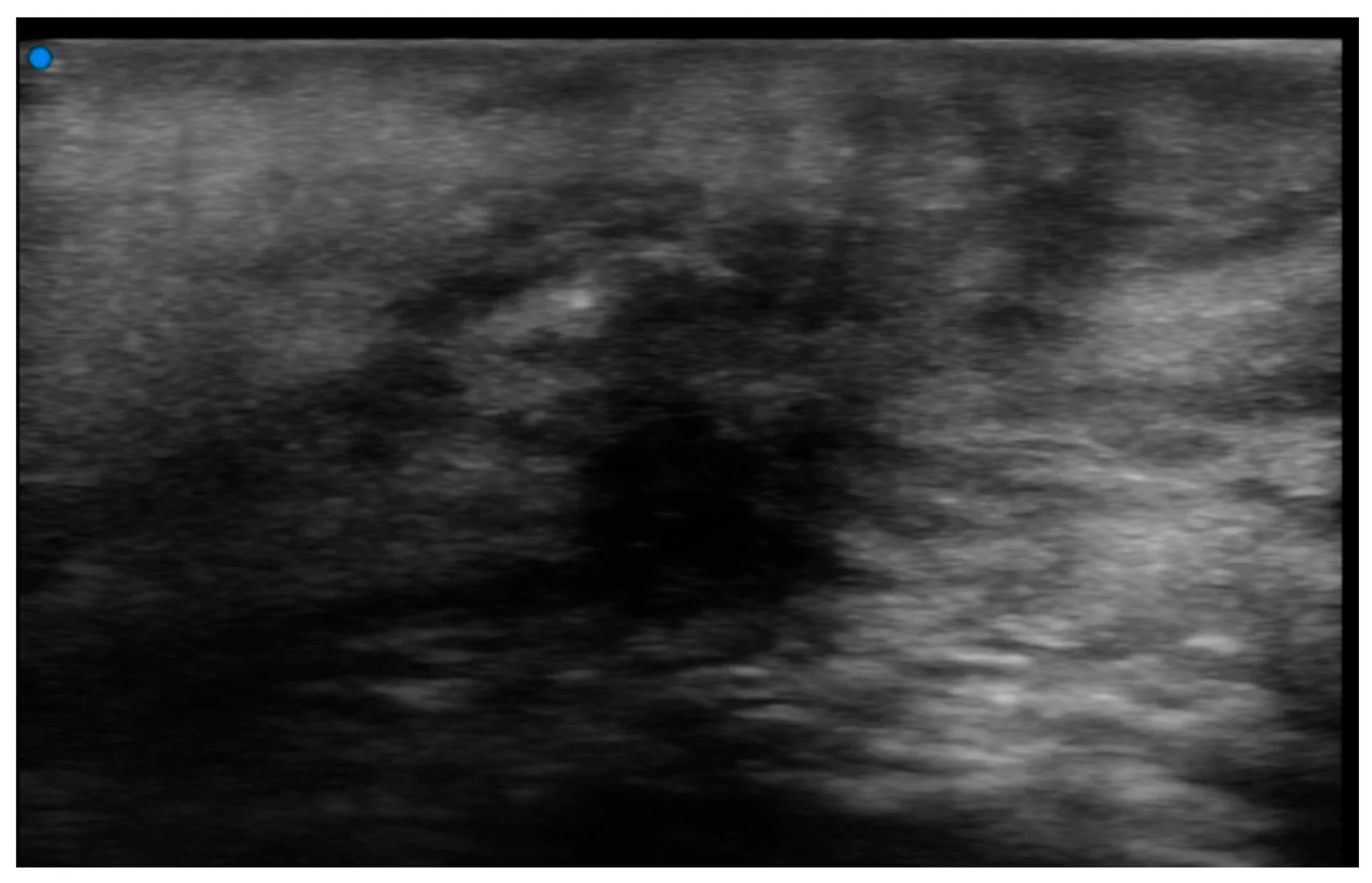
UHFUS appearance of surface discontinuity in primary cutaneous melanoma. Representative ultra-high-frequency ultrasound image showing focal disruption of the superficial echogenic interface overlying the melanoma, classified as surface discontinuity according to the predefined imaging criteria. This sonographic finding was evaluated as a potential imaging correlate of histopathologic ulceration, which is defined microscopically by full-thickness epidermal loss with associated reactive changes in the absence of trauma. UHFUS, ultra-high-frequency ultrasound.

For intraobserver repeatability, all examinations were independently reassessed by both readers after a 4-week washout period, with cases presented in a different randomized order. This washout interval was intended to reduce recall of the initial research assessment and did not constitute a mechanism for eliminating potential prior familiarity derived from the original clinical examination. For each lesion, maximum UHFUS thickness was measured as the greatest perpendicular distance between the superficial and deepest identifiable lesion boundaries, while maximum horizontal diameter was measured parallel to the skin surface [23,29] (Figure 2, Figure 3 and Figure 4).

Surface discontinuity was defined as focal or more extensive disruption of the superficial echogenic interface overlying the lesion and was recorded as present or absent. The deepest sonographically involved anatomical compartment was categorized as dermis, dermal–hypodermal interface, hypodermis, or non-assessable. Lateral and deep lesion boundaries were evaluated separately and classified as well-defined, ill-defined, or non-assessable. Separate perilesional hypoechoic foci were defined as discrete hypoechoic foci separated from the primary lesion by sonographically preserved intervening tissue and were classified as present, absent, or non-assessable. When present, their number, maximum diameter, and minimum distance from the primary lesion were recorded. Lesion morphology was classified as fusiform, nodular, irregular, or other, while internal echostructure was classified as homogeneous or heterogeneous. When technically adequate Color Doppler images were available, vascularity was graded as absent, mild, moderate, or marked and, when detectable, its distribution was categorized as peripheral, intralesional, or mixed.

For variables allowing a non-assessable category, this designation was assigned when the specific feature could not be confidently categorized on the stored UHFUS images despite the examination meeting the overall technical-adequacy criteria for study inclusion. Thus, feature-level non-assessability was distinguished from a technically inadequate examination, which constituted an exclusion criterion at study screening.

For intraobserver repeatability, all examinations were independently reassessed by both readers after a 4-week washout period, with cases presented in a different randomized order.

### 2.4. Histopathologic Reference Standard

Definitive histopathology served as the reference standard. The following variables were collected from the original pathology report and/or reassessed by a dedicated pathologist: Breslow thickness, histological subtype, growth phase, ulceration, microsatellitosis, mitotic rate, Clark level, tumor-infiltrating lymphocytes, regression, lymphovascular invasion, neurotropism, associated nevus component, lateral and deep margin status, and pathological T category [1,3,24].

For the principal radiologic–pathologic comparisons, analyses focused on Breslow thickness, ulceration, depth of invasion, and microsatellitosis [21]. For the depth-of-invasion comparison, histopathologic findings were retrospectively categorized according to the deepest anatomical compartment involved by melanoma: dermis, dermal–hypodermal interface, or hypodermis. This compartment-based categorization was specifically created to allow anatomical comparison with the UHFUS assessment and was not intended to reproduce or substitute for Clark level.

Histopathologic variables without a plausible direct UHFUS equivalent, including tumor-infiltrating lymphocytes, regression, and associated nevus component, were evaluated only as potential modifiers of UHFUS appearance or UHFUS–histopathology thickness discrepancy.

### 2.5. Thickness Categories

In addition to continuous measurements, UHFUS and histopathologic thickness were classified into four predefined strata (≤1.0 mm, >1.0–2.0 mm, >2.0–4.0 mm, and >4.0 mm), corresponding to the Breslow thickness intervals underlying AJCC T1–T4 categorization [2]. This analysis was intended to assess the clinical relevance of UHFUS–histopathology measurement disagreement across established thickness thresholds rather than to assign an imaging-based T category, as pathological T subclassification also incorporates histopathologic ulceration. Agreement was categorized as exact classification, underclassification, or overclassification.

### 2.6. Statistical Analysis

Continuous variables were summarized as mean ± standard deviation or median and interquartile range, according to data distribution, while categorical variables were reported as counts and percentages.

Interobserver reproducibility of continuous UHFUS measurements was assessed using a two-way random-effects, absolute-agreement, single-measure intraclass correlation coefficient (ICC) with 95% confidence intervals. Intraobserver repeatability was assessed separately for each reader using a two-way mixed-effects, absolute-agreement ICC. Interobserver agreement for categorical UHFUS features was evaluated using Cohen’s κ for nominal variables and weighted κ for ordinal variables, together with percentage agreement.

Agreement between UHFUS thickness and histopathologic Breslow thickness was assessed separately for each reader using Lin’s concordance correlation coefficient (CCC) and Bland–Altman analysis. Mean bias and 95% limits of agreement were calculated. For each lesion, thickness discrepancy was defined as:

*ΔThickness = UHFUS thickness − histopathologic Breslow thickness.*

Positive values indicated UHFUS overestimation and negative values underestimation. Absolute measurement error was also calculated.

The association of surface discontinuity with histopathologic ulceration and of perilesional hypoechoic foci with microsatellitosis was evaluated using sensitivity, specificity, positive predictive value, negative predictive value, and overall accuracy, with exact binomial 95% confidence intervals. Agreement between UHFUS-derived and histopathologic thickness strata was assessed separately for each reader using complete 4 × 4 contingency tables, weighted κ coefficients with 95% confidence intervals, exact agreement, and the proportions of underclassified and overclassified lesions. As a prespecified secondary analysis, diagnostic performance for classification below versus at or above the 0.8 mm threshold was assessed separately for each reader using sensitivity, specificity, positive predictive value, negative predictive value, overall accuracy, and exact binomial 95% confidence intervals.

Associations between ΔThickness and predefined histopathologic variables were initially explored using univariable analyses and subsequently evaluated using multivariable linear regression when supported by the distribution of the outcome and the number of observations. Regression assumptions were assessed, and robust estimates were used in the presence of relevant heteroscedasticity or influential observations.

Missing data were not imputed, and analyses were performed using available cases, with the denominator reported for each analysis. Statistical analyses were performed using IBM SPSS Statistics for Windows, version 32.0 (IBM Corp., Armonk, NY, USA). All tests were two-sided, and *p* values < 0.05 were considered statistically significant.

## 3. Results

### 3.1. Study Population

During the study period, 357 treatment-naïve patients with 357 clinically suspicious melanocytic skin lesions underwent preoperative UHFUS. Overall, 37 lesions were excluded: 10 because of insufficient image quality for reliable assessment and 27 because definitive histopathology did not confirm melanoma. The final analytic cohort therefore comprised 320 histologically confirmed primary cutaneous melanomas in 320 patients. Median patient age was 58 years (IQR, 45–70), and 170 patients (53.1%) were male. The trunk was the most frequent site (*n* = 128, 40.0%), followed by the lower extremities (*n* = 80, 25.0%), upper extremities (*n* = 64, 20.0%), and head and neck (*n* = 48, 15.0%). Superficial spreading melanoma was the predominant histological subtype (*n* = 183, 57.2%). Histopathologic ulceration was present in 55 lesions (17.2%), microsatellitosis in 16 (5.0%), regression in 80 (25.0%), and an associated nevus component in 90 (28.1%). Median histopathologic Breslow thickness was 1.1 mm. Baseline clinical and histopathologic characteristics are summarized in Table 1.

### 3.2. Interobserver Reproducibility

Median UHFUS thickness was 1.2 mm (IQR, 0.7–2.1) for both readers. Interobserver reproducibility was excellent for maximum tumor thickness, with an ICC of 0.96 (95% CI, 0.95–0.97), and for maximum horizontal diameter, with an ICC of 0.94 (95% CI, 0.92–0.95).

Among categorical features, the highest interobserver agreement was observed for lesion morphology (κ = 0.87; 95% CI, 0.83–0.91), deepest involved compartment (weighted κ = 0.85; 95% CI, 0.81–0.89), and surface discontinuity (weighted κ = 0.83; 95% CI, 0.78–0.88). Agreement was lower, although still substantial, for internal echostructure (κ = 0.77; 95% CI, 0.72–0.82), lateral boundary definition (weighted κ = 0.75; 95% CI, 0.69–0.81), and deep boundary definition (weighted κ = 0.72; 95% CI, 0.66–0.78). Separate perilesional hypoechoic foci showed 97% interobserver agreement, with a κ of 0.84 (95% CI, 0.73–0.94). Color Doppler assessment was technically adequate in 272 lesions. Reader 1 classified vascularity as absent in 74 (27.2%), mild in 91 (33.5%), moderate in 72 (26.5%), and marked in 35 (12.9%); corresponding values for Reader 2 were 77 (28.3%), 88 (32.4%), 70 (25.7%), and 37 (13.6%), respectively. Interobserver agreement for vascularity grade was substantial (κ = 0.78; 95% CI, 0.73–0.83). Among lesions with detectable vascularity, distribution was predominantly peripheral or mixed, with substantial interobserver agreement for vascularity distribution (κ = 0.75; 95% CI, 0.69–0.81).

Intraobserver repeatability of tumor thickness measurements was excellent for both readers, with an ICC of 0.97 (95% CI, 0.96–0.98). Detailed reproducibility results are reported in Table 2.

### 3.3. Agreement Between UHFUS and Histopathologic Thickness

**Figure 5:**
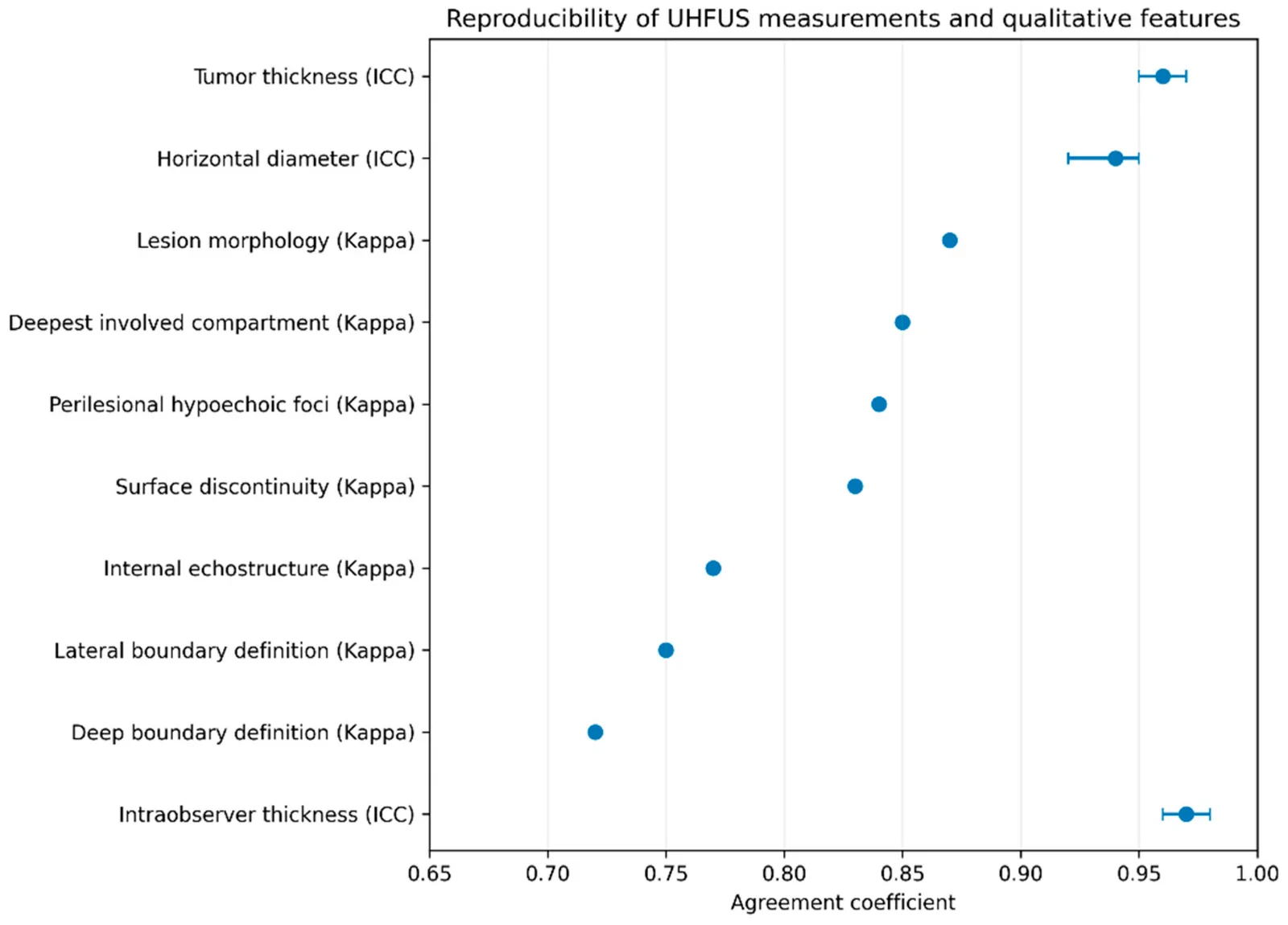
Reproducibility of UHFUS measurements and qualitative imaging features. Point estimates of interobserver reproducibility are shown for continuous and categorical UHFUS variables. Intraclass correlation coefficients (ICC) are reported for continuous measurements and κ coefficients for categorical features. Where available, 95% confidence intervals are shown. Intraobserver repeatability is additionally reported for tumor-thickness measurements.

Agreement between UHFUS and histopathologic Breslow thickness was excellent for both readers, with Lin’s concordance correlation coefficients of 0.95 (95% CI, 0.94–0.96) for Reader 1 and 0.95 (95% CI, 0.94–0.96) for Reader 2 (Figure 5).

Bland–Altman analysis showed a small positive bias of +0.12 mm for Reader 1, with 95% limits of agreement from −0.34 to +0.58 mm, and +0.10 mm for Reader 2, with limits from −0.36 to +0.56 mm. Median absolute error was approximately 0.14 mm. UHFUS measurements more frequently overestimated than underestimated histopathologic thickness.

### 3.4. Agreement Across Thickness Categories

Categorical agreement with histopathologic Breslow-thickness strata was assessed separately for the two readers. Histopathologically, 145 melanomas (45.3%) had a Breslow thickness ≤ 1.0 mm, 95 (29.7%) measured >1.0–2.0 mm, 55 (17.2%) measured >2.0–4.0 mm, and 25 (7.8%) measured >4.0 mm. For Reader 1, UHFUS and histopathology assigned 288 of 320 lesions (90.0%) to the same thickness category. Thirteen lesions (4.1%) were underclassified and 19 (5.9%) were overclassified by UHFUS. The corresponding linearly weighted κ was 0.90 (95% CI, 0.87–0.93). For Reader 2, exact categorical agreement was observed in 293 of 320 lesions (91.6%), with 11 lesions (3.4%) underclassified and 16 lesions (5.0%) overclassified. The corresponding linearly weighted κ was 0.91 (95% CI, 0.88–0.94). For both readers, most discordant classifications involved migration between adjacent thickness strata rather than displacement across multiple categories. The complete cross-tabulations are reported in Table 3 and Table 4.

Stratum-specific exact agreement was relatively consistent across the four histopathologic thickness categories. For Reader 1, exact agreement was 89.7% (130/145) for lesions ≤ 1.0 mm, 89.5% (85/95) for lesions > 1.0–2.0 mm, 90.9% (50/55) for lesions > 2.0–4.0 mm, and 92.0% (23/25) for lesions > 4.0 mm. Corresponding values for Reader 2 were 91.7% (133/145), 91.6% (87/95), 90.9% (50/55), and 92.0% (23/25), respectively. In the prespecified secondary analysis using the 0.8 mm threshold, 211 lesions had a histopathologic Breslow thickness ≥0.8 mm and 109 had a Breslow thickness < 0.8 mm. For Reader 1, UHFUS classified 303 of 320 lesions (94.7%) on the same side of the threshold as histopathology. Eight lesions were classified as <0.8 mm by UHFUS despite a histopathologic Breslow thickness ≥ 0.8 mm, whereas nine were classified as ≥0.8 mm by UHFUS despite a histopathologic Breslow thickness < 0.8 mm. Sensitivity, specificity, positive predictive value, negative predictive value, and overall accuracy for identifying lesions with a histopathologic Breslow thickness ≥ 0.8 mm were 96.2% (95% CI, 92.7–98.3%), 91.7% (95% CI, 84.9–96.2%), 95.8% (95% CI, 92.1–98.0%), 92.6% (95% CI, 85.9–96.7%), and 94.7% (95% CI, 91.6–96.9%), respectively. For Reader 2, 305 of 320 lesions (95.3%) were classified on the same side of the threshold as histopathology, with seven downward and eight upward threshold crossings. Corresponding sensitivity, specificity, positive predictive value, negative predictive value, and overall accuracy were 96.7% (95% CI, 93.3–98.7%), 92.7% (95% CI, 86.0–96.8%), 96.2% (95% CI, 92.7–98.4%), 93.5% (95% CI, 87.1–97.4%), and 95.3% (95% CI, 92.4–97.4%), respectively.

### 3.5. Surface Discontinuity and Histopathologic Ulceration

Histopathologic ulceration was present in 55 melanomas (17.2%). Surface discontinuity was identified in 57 lesions by Reader 1 and in 55 by Reader 2. For Reader 1, surface discontinuity showed a sensitivity of 87.3% (95% CI, 75.5–94.7%), specificity of 96.6% (95% CI, 93.7–98.4%), positive predictive value of 84.2% (95% CI, 72.1–92.5%), negative predictive value of 97.3% (95% CI, 94.6–98.9%), and overall accuracy of 95.0% (95% CI, 92.0–97.1%) for histopathologic ulceration. For Reader 2, the corresponding sensitivity was 85.5% (95% CI, 73.3–93.5%), specificity 97.0% (95% CI, 94.1–98.7%), positive predictive value 85.5% (95% CI, 73.3–93.5%), negative predictive value 97.0% (95% CI, 94.1–98.7%), and overall accuracy 95.0% (95% CI, 92.0–97.1%).

### 3.6. Depth and Satellite Foci

The deepest sonographically involved compartment showed exact agreement with the corresponding histopathologic anatomical-compartment category in 90.0% of lesions, with a weighted κ of 0.88. Microsatellitosis was present histopathologically in 16 of 320 melanomas (5.0%). The analysis of separate perilesional hypoechoic foci was performed in the full study cohort (*n* = 320), and such foci were identified in 18 lesions (5.6%). Among lesions with detectable perilesional hypoechoic foci, the median number of foci was 1 (IQR, 1–2), the median maximum focus diameter was 0.9 mm (IQR, 0.6–1.4 mm), and the median minimum distance from the primary lesion was 1.6 mm (IQR, 0.9–2.7 mm). Perilesional hypoechoic foci showed a sensitivity of 62.5% (95% CI, 35.4–84.8%), specificity of 97.4% (95% CI, 94.9–98.9%), positive predictive value of 55.6% (95% CI, 30.8–78.5%), negative predictive value of 98.0% (95% CI, 95.7–99.3%), and overall accuracy of 95.6% (95% CI, 92.8–97.6%) for histopathologic microsatellitosis.

### 3.7. Histopathologic Determinants of Thickness Discrepancy

Median ΔThickness was +0.10 mm (IQR, −0.02 to +0.22 mm). On univariable analysis, UHFUS overestimation was significantly greater in melanomas with an associated nevus component and in those with increased tumor-infiltrating lymphocytes (TILs), whereas regression, ulceration, and histological subtype were not significantly associated with thickness discrepancy.

**Figure 6:**
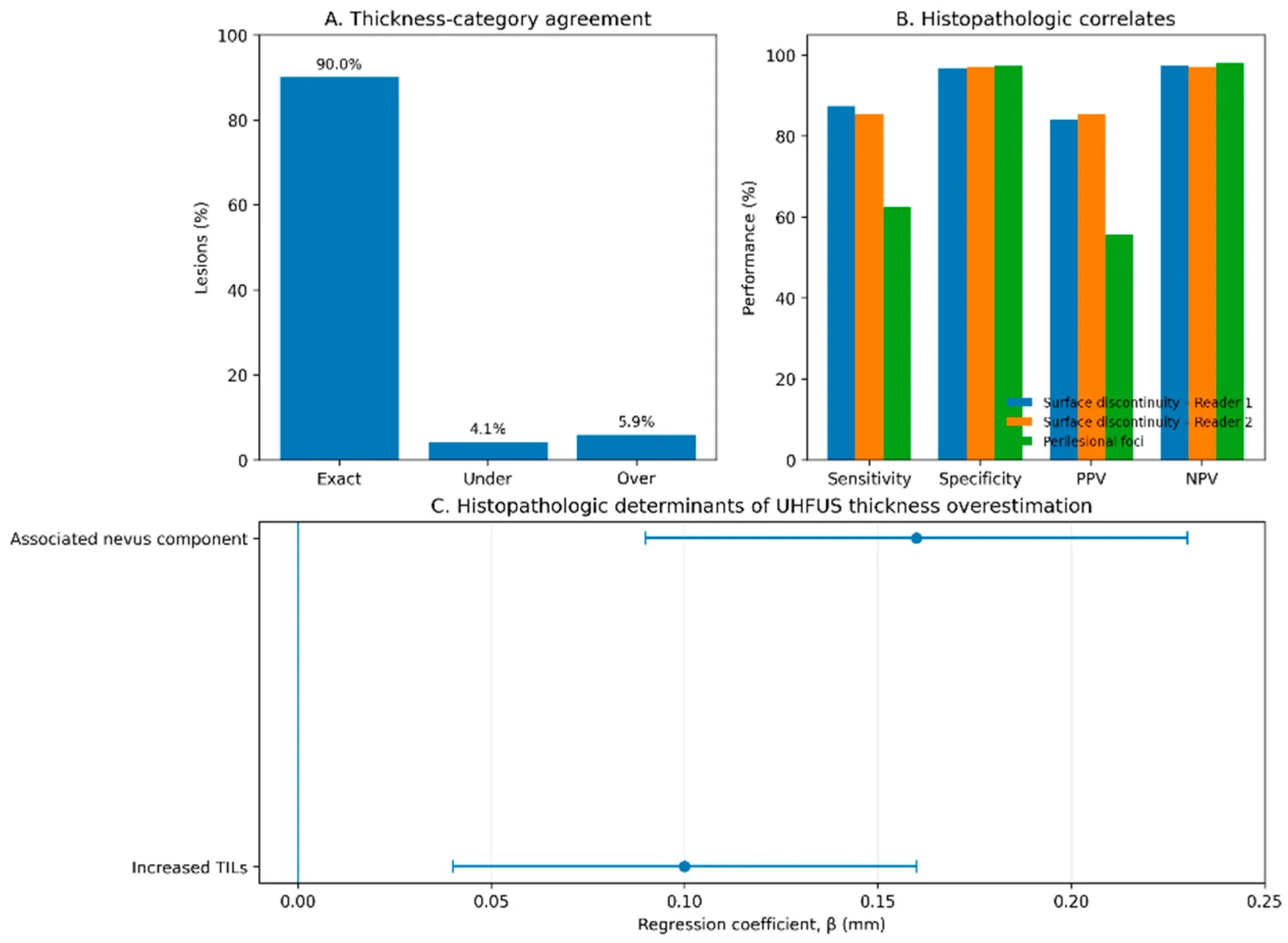
Radiologic–pathologic concordance and secondary analyses: (**A**) Agreement between UHFUS and histopathology across predefined melanoma thickness categories. (**B**) Diagnostic performance of sonographic surface discontinuity for histopathologic ulceration and of separate perilesional hypoechoic foci for microsatellitosis. (**C**) Multivariable linear-regression estimates for histopathologic variables independently associated with greater UHFUS thickness overestimation. PPV, positive predictive value; NPV, negative predictive value; TILs, tumor-infiltrating lymphocytes.

At multivariable analysis, both the presence of an associated nevus component (β = +0.16 mm; 95% CI, +0.09 to +0.23; *p* < 0.001) and increased TILs (β = +0.10 mm; 95% CI, +0.04 to +0.16; *p* = 0.002) remained independently associated with greater UHFUS overestimation (Figure 6).

## 4. Discussion

The present study supports the reproducibility of a structured UHFUS assessment of treatment-naïve primary cutaneous melanoma in a dedicated setting. The principal finding is not simply the close relationship between sonographic and histopathologic thickness, which has already been demonstrated in previous HFUS and UHFUS studies, but the consistency of measurements between experienced readers and the reproducibility of a broader set of predefined morphological and anatomical features. These findings extend previous evidence by evaluating UHFUS within a comparatively large cohort and by combining measurement agreement, reader reproducibility, categorical thickness assessment, and selected radiologic–pathologic comparisons within a single standardized framework.

Agreement between UHFUS and histopathologic Breslow thickness was high for both readers, although the two measurements should not be regarded as geometrically equivalent. UHFUS thickness is measured from the superficial to the deepest identifiable sonographic lesion boundary, whereas Breslow thickness is based on histopathologically defined landmarks and specifically quantifies invasive melanoma [3,30]. Histopathologic processing, tissue composition, and residual compression during scanning may therefore affect the two measurements differently. The small positive UHFUS–histopathology bias observed in this cohort should consequently be interpreted as the combined effect of several potential sources of discrepancy rather than as evidence of a single dominant mechanism. Previous melanoma studies and the recent meta-analysis have likewise demonstrated close correspondence between sonographic and histopathologic thickness, while also showing heterogeneity related to ultrasound frequency, study population, and measurement methodology [23,31,32,33]. The present findings should therefore be regarded as an extension of this literature rather than as evidence that UHFUS and Breslow thickness are interchangeable measurements.

Categorical agreement should also be interpreted with caution. Although agreement across the predefined Breslow-thickness strata was high for both readers, both percentage agreement and weighted κ are influenced by the marginal distribution of lesions across categories and cannot be considered distribution-independent measures of performance. In the present cohort, stratum-specific agreement remained relatively consistent across the four histopathologic thickness groups, but the same aggregate agreement cannot necessarily be expected in populations with different Breslow-thickness distributions. This is particularly relevant when measurements are close to predefined category boundaries, where small absolute differences may alter category assignment. Histopathologic staging therefore remains the reference standard, and UHFUS-derived thickness strata should not be interpreted as imaging-based pathological T categories [3,34]. Previous prospective work by Varga et al. [31] similarly demonstrated high agreement for thickness-based classification using optically guided 33 MHz HFUS, with a κ of 0.858 in 101 patients.

The structured assessment of features beyond thickness represents an additional aspect of the present study. Morphology, surface discontinuity, lesion boundaries, deepest involved anatomical compartment, and perilesional findings showed substantial reproducibility between readers, suggesting that UHFUS assessment need not be restricted to a single linear measurement. This concept is consistent with previous work emphasizing the ability of dermatologic HFUS and UHFUS to characterize lesion architecture, anatomical relationships, and surrounding tissues [23,29,35,36]. The deepest involved compartment should not be interpreted as a sonographic equivalent of Clark level, but rather as an anatomical description of the deepest recognizable skin compartment involved by the lesion. Similarly, surface discontinuity represents a macroscopic sonographic feature that may correspond to, but is not synonymous with, histopathologic ulceration, which remains a microscopic criterion relevant to pathological staging [9,25,34].

Separate perilesional hypoechoic foci provide another example of the distinction between imaging appearance and histopathologic definition. Their high specificity for histopathologic microsatellitosis suggests that a discrete hypoechoic focus separated from the primary lesion by preserved tissue may represent a meaningful preoperative finding. Conversely, their more limited sensitivity indicates that the absence of a visible focus cannot exclude microscopic disease. This is consistent with the intrinsic difference in spatial scale between imaging and histopathology and with previous observations regarding the capabilities and limitations of high-frequency ultrasound in the assessment of cutaneous malignancies [21,28]. Given the limited number of melanomas with microsatellitosis in the present cohort, this finding should nevertheless be regarded as exploratory and requires confirmation in larger dedicated populations.

The association of increased tumor-infiltrating lymphocytes and an associated nevus component with greater UHFUS thickness discrepancy provides a possible explanation for part of the residual sonographic–histopathologic difference. These associations, however, should not be interpreted causally. UHFUS depicts the overall acoustic abnormality in vivo, whereas Breslow thickness specifically measures invasive malignant melanocytes according to microscopic criteria. Inflammatory infiltrates or nevomelanocytic components may therefore contribute differently to the sonographically visible lesion and to the histopathologic measurement. At the same time, the present data do not allow the independent contributions of lesion composition, differing measurement landmarks, histopathologic processing, or residual probe compression to be quantified. Accordingly, the observed bias should be interpreted as a composite measurement difference rather than being attributed to any single histological or technical factor.

The reproducibility observed in this study must also be considered in relation to operator expertise and standardization. Very-high- and ultra-high-frequency imaging are particularly dependent on appropriate probe positioning, minimal compression, correct identification of lesion boundaries, and familiarity with normal and pathological skin anatomy. The present results were obtained by highly experienced radiologists in a dedicated setting and should not be assumed to transfer directly to less experienced operators or institutions. Expertise and standardized acquisition and interpretation should therefore be regarded as integral components of the technique rather than as incidental characteristics of the present study. Future multicenter investigations should include readers with different levels of experience and specifically address acquisition reproducibility, the learning curve, and competency requirements for melanoma UHFUS.

Several features strengthen the present study. The cohort is larger than those included in most individual studies summarized in the recent meta-analysis [21], and all included melanomas were examined before biopsy or local treatment. This treatment-naïve design avoids distortion related to partial lesion removal, inflammation, hemorrhage, or post-biopsy architectural change and permits comparison between the intact in vivo lesion and subsequent definitive histopathology. The study also evaluates agreement rather than correlation alone and extends the assessment beyond tumor thickness to predefined morphological and anatomical variables.

Several limitations should nevertheless be acknowledged. The retrospective single-center design and the use of highly experienced readers restrict immediate generalizability. The same radiologists who performed the original clinical UHFUS examinations subsequently participated in the retrospective image-review phase; although the formal research readings were conducted independently, in randomized anonymized order, and without access to definitive histopathologic results, complete absence of prior familiarity with individual lesions cannot be guaranteed. In addition, exclusion of technically inadequate examinations means that the reported reproducibility and agreement estimates apply to technically assessable UHFUS studies and may somewhat overestimate performance in an unselected clinical population. Histopathology itself is affected by fixation, processing, orientation, and tissue contraction and does not represent an identical physical measurement of the in vivo lesion. The small number of cases with microsatellitosis limits the precision of the corresponding diagnostic-performance estimates. Furthermore, because lesions not ultimately confirmed as melanoma were excluded from the analytic cohort, the present study cannot estimate the diagnostic accuracy of UHFUS for differentiating melanoma from benign or other malignant cutaneous lesions. Finally, this retrospective non-interventional study was not designed to determine whether UHFUS modifies excision margins, sentinel lymph-node management, systemic treatment selection, or patient outcomes; its demonstrated role is limited to reproducible preoperative characterization of the intact primary lesion, and any incremental clinical utility requires prospective evaluation.

## 5. Conclusions

UHFUS provides a highly reproducible preoperative assessment of treatment-naïve primary cutaneous melanoma, with excellent interobserver and intraobserver reproducibility for tumor-thickness measurements and strong concordance with histopathologic Breslow thickness. Reproducibility also extends to selected morphological and anatomical features, including lesion morphology, surface discontinuity, boundary definition, deepest involved compartment, and perilesional findings. However, the observed categorical agreement across Breslow-thickness strata should be interpreted in the context of the thickness distribution of the present cohort and should not be considered a distribution-independent measure of performance.

Surface discontinuity showed a strong association with histopathologic ulceration, whereas separate perilesional hypoechoic foci demonstrated high specificity but limited sensitivity for microsatellitosis; the latter finding should be regarded as exploratory because of the small number of positive cases. Increased tumor-infiltrating lymphocytes and the presence of an associated nevus component were independently associated with greater UHFUS–histopathology thickness discrepancy, suggesting that lesion composition may contribute to measurement differences, although the relative contributions of tissue composition, differing measurement landmarks, histopathologic processing, and residual probe compression cannot be determined from the present study.

UHFUS should therefore be regarded as a complementary preoperative imaging method for characterization of the intact primary lesion rather than as a substitute for histopathologic staging or as an independently validated decision-making tool. The present retrospective, non-interventional study does not establish that UHFUS modifies excision margins, sentinel lymph-node management, systemic treatment selection, or patient outcomes. Prospective multicenter studies involving readers with different levels of experience are required to confirm reproducibility across clinical settings, define training requirements, and determine whether the additional preoperative information provided by UHFUS offers incremental clinical utility.

## Figures and Tables

**Table 1:** Baseline clinical and histopathologic characteristics of the study cohort. Data are presented as *n* (%) unless otherwise indicated. IQR, interquartile range.

Characteristic	Overall Cohort (*n* = 320)
Age, years, median (IQR)	58 (45–70)
Male sex, *n* (%)	170 (53.1)
**Anatomical site, *n* (%)**	
Trunk	128 (40.0)
Lower extremities	80 (25.0)
Upper extremities	64 (20.0)
Head and neck	48 (15.0)
**Histological subtype, *n* (%)**	
Superficial spreading melanoma	183 (57.2)
Nodular melanoma	43 (13.4%)
Lentigo maligna melanoma	51 (15.9%)
Acral lentiginous melanoma	11 (3.4%)
Other	32 (10%)
Histopathologic Breslow thickness strata, *n* (%) <1.0 mm: 1.0–2.0 mm: 2.0–4.0 mm: 4.0 mm:	145 (45.3%) 95 (29.7%) 55 (17.2%) 25 7.8%
Histopathologic Breslow thickness strata, *n* (%) 1.0–2.0 mm: 2.0–4.0 mm: 4.0 mm:	≤1.0 mm: 145 (45.3%) 95 (29.7%) 55 (17.2%) 25 (7.8%)
Ulceration, *n* (%)	55 (17.2)
Microsatellitosis, *n* (%)	16 (5.0)
Regression, *n* (%)	80 (25.0)
Associated nevus component, *n* (%)	90 (28.1)

**Table 2:** Interobserver reproducibility and intraobserver repeatability of UHFUS measurements and qualitative imaging features. ICC, intraclass correlation coefficient; CI, confidence interval; κ, kappa coefficient; UHFUS, ultra-high-frequency ultrasound. Percentage agreement is additionally reported for separate perilesional hypoechoic foci.

UHFUS Parameter	Agreement Measure	Estimate	95% CI/Agreement
**Continuous measurements—interobserver reproducibility**			
Maximum tumor thickness	ICC	0.96	0.95–0.97
Maximum horizontal diameter	ICC	0.94	0.92–0.95
**Categorical features—interobserver agreement**			
Lesion morphology	Cohen’s κ	0.87	0.83–0.91
Deepest involved compartment	Weighted κ	0.85	0.81–0.89
Surface discontinuity	Weighted κ	0.83	0.78–0.88
Separate perilesional hypoechoic foci	Cohen’s κ	0.84	0.73–0.94
Internal echostructure	Cohen’s κ	0.77	0.72–0.82
Lateral boundary definition	Weighted κ	0.75	0.69–0.81
Deep boundary definition	Weighted κ	0.72	0.66–0.86
**Intraobserver repeatability—tumor thickness**			
Reader 1	ICC	0.97	0.96–0.98
Reader 2	ICC	0.97	0.96–0.98

**Table 3:** Cross-tabulation of UHFUS-derived thickness categories versus histopathologic Breslow-thickness strata for Reader 1. Rows represent histopathologic Breslow-thickness strata and columns represent UHFUS-derived thickness categories. Cells on the main diagonal represent exact agreement; cells below the diagonal represent UHFUS underclassification and cells above the diagonal represent UHFUS overclassification.

Histopathologic Breslow Stratum	UHFUS ≤ 1.0 mm	UHFUS > 1.0–2.0 mm	UHFUS > 2.0–4.0 mm	UHFUS > 4.0 mm	Total
≤1.0 mm	130	13	2	0	145
>1.0–2.0 mm	6	85	4	0	95
>2.0–4.0 mm	0	5	50	0	55
>4.0 mm	0	0	2	23	25
Total	136	103	58	23	320

**Table 4:** Cross-tabulation of UHFUS-derived thickness categories versus histopathologic Breslow-thickness strata for Reader 2. Rows represent histopathologic Breslow-thickness strata and columns represent UHFUS-derived thickness categories. Cells on the main diagonal represent exact agreement; cells below the diagonal represent UHFUS underclassification and cells above the diagonal represent UHFUS overclassification.

Histopathologic Breslow Stratum	UHFUS ≤ 1.0 mm	UHFUS > 1.0–2.0 mm	UHFUS > 2.0–4.0 mm	UHFUS > 4.0 mm	Total
≤1.0 mm	133	11	1	0	145
>1.0–2.0 mm	5	87	3	0	95
>2.0–4.0 mm	0	4	50	1	55
>4.0 mm	0	0	2	23	25
Total	138	102	56	24	320

## Data Availability

Data available on demand to the Corresponding Author due to privacy restrictions.

## Supplementary Materials

The following supporting information can be downloaded at: https://www.mdpi.com/article/10.3390/diagnostics16203416/s1, Table S1: STARD 2015 checklist—diagnostic-accuracy components; Table S2: GRRAS checklist—reliability and agreement components; Table S3: Cross-tabulations for secondary diagnostic-accuracy analyses.
