# Supplementary material for: Beyond Breslow Thickness: Reproducibility and Histopathologic Concordance of Ultra-High-Frequency Ultrasound in Primary Cutaneous Melanoma
Source: Diagnostics (Basel). 2026 Oct 20;16(20):3416. doi: 10.3390/diagnostics16203416 (PMC13703478; doi:10.3390/diagnostics16203416)
Supplement: Supplementary file 1 [file diagnostics-16-03416-s001.zip › diagnostics-4550105-supplementary.pdf]

Supplementary Reporting-Guideline Checklists

Supplementary Table S1. STARD 2015 checklist - diagnostic-accuracy components

Item	Reporting recommendation	Where reported / manuscript content
1	Identify the article as a study of diagnostic accuracy, using at least one measure of accuracy.	Abstract: the diagnostic-accuracy component is secondary to the primary reproducibility/agreement aims; sensitivity/specificity and predictive performance are summarized in the Results portion of the abstract. The title emphasizes reproducibility and histopathologic concordance rather than diagnostic accuracy.
2	Provide a structured summary of study design, methods, results, and conclusions.	Abstract: structured Background, Methods, Results, and Conclusions.
3	Describe the scientific and clinical background, including the intended use and clinical role of the index test.	Introduction; Discussion; Conclusions. UHFUS is framed as a complementary preoperative characterization method, not a substitute for histopathology or an independently validated decision-making tool.
4	State the study objectives and hypotheses.	End of Introduction: primary objectives are reproducibility of UHFUS thickness and agreement with Breslow thickness; secondary objectives include reproducibility of morphological features, radiologic-pathologic associations, categorical thickness agreement, and determinants of thickness discrepancy.
5	State whether data collection was planned before or after the index test and reference standard were performed.	Methods §2 and §2.1: retrospective, non-interventional analysis of UHFUS examinations and data previously acquired during routine clinical practice.
6	Describe participant eligibility criteria.	Methods §2.1: treatment-naïve clinically suspicious melanocytic lesions; technically adequate UHFUS; subsequent definitive histopathology; exclusion criteria specified.
7	Describe how potentially eligible participants were identified.	Methods §2.1: consecutive treatment-naïve patients referred by dermatologists for preoperative UHFUS assessment of a clinically suspicious melanocytic lesion.
8	State the setting, location, and dates of participant identification.	Methods §2.1: University Hospital 'Luigi Vanvitelli', Naples, Italy; January 2025-July 2026.
9	State whether the participant series was consecutive, random, or convenience based.	Methods §2.1: consecutive patients.
10a	Describe the index test in sufficient detail to allow replication.	Methods §§2.2-2.3: Vevo 3100 system, 48-70 MHz linear transducer, longitudinal/transverse acquisition, coupling gel/stand-off, B-mode and Color Doppler, reader procedures, and measurement definitions.
10b	Describe the reference standard in sufficient detail to allow replication.	Methods §2.4: definitive histopathology as reference standard; histopathologic variables collected from the original pathology report and/or reassessed by a dedicated pathologist.
11	Explain the rationale for choosing the reference standard.	Introduction and Methods §2.4: definitive histopathology is the reference standard for diagnosis/staging of primary melanoma and is used for the radiologic-pathologic comparisons.
12a	Define index-test positivity cut-offs or result categories and distinguish prespecified from exploratory choices.	Methods §§2.3, 2.5, and 2.6: predefined surface discontinuity and perilesional-foci criteria; four predefined thickness strata; prespecified secondary 0.8-mm threshold analysis.
12b	Define reference-standard positivity cut-offs or result categories and distinguish prespecified from exploratory choices.	Methods §§2.4-2.5: histopathologic ulceration and microsatellitosis; Breslow-thickness strata; histopathologic anatomical-compartment categories used for the depth comparison.
13a	State what clinical information and reference-standard results were available to readers of the index test.	Methods §§2.2-2.3: readers assessed cases separately, without access to each other's assessments or definitive histopathologic results; cases were randomized/anonymized. Prior familiarity from participation in the original clinical examinations could not be completely excluded.

Item	Reporting recommendation	Where reported / manuscript content
13b	State what clinical information and index-test results were available to assessors of the reference standard.	Not reported in the manuscript: the manuscript does not state whether the pathologist/reference-standard assessor was blinded to UHFUS findings.
14	Describe methods for estimating or comparing measures of diagnostic accuracy.	Methods §2.6: sensitivity, specificity, PPV, NPV, overall accuracy, and exact binomial 95% CIs; reader-specific analyses where applicable.
15	Describe how indeterminate index-test or reference-standard results were handled.	Methods §§2.1, 2.3, and 2.6: technically inadequate examinations were excluded at eligibility screening; non-assessable imaging categories were permitted for selected qualitative features; available-case denominators were used where applicable.
16	Describe how missing data on the index test and reference standard were handled.	Methods §2.6: missing data were not imputed; analyses were performed using available cases, with denominators reported for each analysis.
17	Describe analyses of variability in diagnostic accuracy and distinguish prespecified from exploratory analyses.	Methods §2.6 and Results §§3.4-3.6: diagnostic-performance analyses are presented separately by reader when applicable; the 0.8-mm threshold analysis is prespecified; the microsatellitosis analysis is interpreted as exploratory because of the small number of positive cases.
18	State the intended sample size and how it was determined.	No formal a priori sample-size calculation is reported. The manuscript states that consecutive eligible patients from the defined retrospective study period were screened and that 320 histologically confirmed melanomas formed the final analytic cohort.
19	Report participant flow, preferably with a diagram.	Methods §2.1, Figure 1, and Results §3.1: 357 lesions screened; 10 excluded for technically inadequate UHFUS; 27 excluded because histopathology did not confirm melanoma; 320 melanomas included.
20	Report baseline demographic and clinical characteristics.	Results §3.1 and Table 1.
21a	Report the distribution of severity of disease in participants with the target condition.	Results §3.1 and Table 1: Breslow-thickness distribution/strata, ulceration, microsatellitosis, regression, histological subtype, and associated nevus component.
21b	Report alternative diagnoses in participants without the target condition.	Methods §2.1: among the 27 non-melanoma exclusions, final diagnoses were melanocytic nevi (n=15), pigmented basal cell carcinomas (n=5), dermatofibromas (n=2), and other cutaneous lesions (n=5).
22	Report the interval and any clinical interventions between index test and reference standard.	Methods §2.1: UHFUS was performed before any biopsy, partial excision, or local treatment of the target lesion. The exact UHFUS-to-definitive-histopathology interval is not reported.
23	Provide a cross-tabulation of the index-test results by the reference-standard results.	Results §3.4: complete reader-specific 4x4 cross-tabulations for UHFUS versus histopathologic thickness strata. The prespecified 0.8-mm threshold analysis is reported in Results §3.4. Supplementary Table S3 provides 2x2 cross-tabulations for the 0.8-mm threshold, ulceration, and microsatellitosis analyses.
24	Report estimates of diagnostic accuracy and measures of statistical uncertainty.	Results §§3.4-3.6: sensitivity, specificity, PPV, NPV, overall accuracy, and 95% CIs are reported for the 0.8-mm threshold, ulceration, and microsatellitosis analyses.
25	Report adverse events related to the index test or reference standard.	Not applicable to the research protocol: the study was retrospective and non-interventional, and no additional research examinations or procedures were performed.
26	Discuss study limitations, including sources of potential bias, statistical uncertainty, and generalisability.	Discussion/Limitations: retrospective single-center design, experienced readers, possible prior familiarity, exclusion of technically inadequate examinations, limited microsatellitosis count, and distribution dependence of categorical agreement.
27	Discuss implications for practice, including intended use and clinical role of the index test.	Discussion and Conclusions: UHFUS is presented as complementary preoperative characterization; the study does not establish that it changes margins, sentinel-node management, systemic treatment, or outcomes.
28	Provide registration details, or state that the study was not registered.	No prospective diagnostic-study registration is reported; the investigation is a retrospective observational study.

Item	Reporting recommendation	Where reported / manuscript content
29	State where the full study protocol can be accessed.	No public protocol repository is reported in the manuscript. Ethics approval details are provided in Methods and the Institutional Review Board Statement.
30	Report sources of funding and the role of funders.	Funding statement: the research received no external funding.

Supplementary Table S2. GRRAS checklist - reliability and agreement components

Item	Reporting recommendation	Where reported / manuscript content
1	Identify in the title or abstract that interrater/intrarater reliability or agreement was investigated.	Title and Abstract: reproducibility and histopathologic concordance are explicit; interobserver and intraobserver measures are summarized.
2	Name and describe the diagnostic or measurement device.	Introduction and Methods §2.2: UHFUS; Vevo 3100 system; 48-70 MHz linear transducer.
3	Specify the subject population of interest.	Methods §2.1: treatment-naïve, histologically confirmed primary cutaneous invasive melanomas assessed preoperatively.
4	Specify the rater population of interest.	Methods §§2.2-2.3: two experienced radiologists; the manuscript describes their dermatologic ultrasound/HFUS-UHFUS experience and their role in acquisition and retrospective review.
5	Summarize prior evidence on reliability/agreement and explain the rationale for the study.	Introduction: prior melanoma ultrasound literature is summarized; the rationale is to extend assessment beyond thickness to a structured quantitative and morphological UHFUS evaluation.
6	Explain sample-size choice and state the numbers of raters, subjects/objects, and replicate observations.	Methods/Results: two readers and 320 melanomas are reported; repeat readings after a 4-week washout are described. No formal a priori reliability sample-size calculation is reported.
7	Describe the sampling method.	Methods §2.1: consecutive eligible patients from the defined retrospective study period.
8	Describe the measurement/rating process, including repeat interval, information available, and blinding.	Methods §§2.2-2.3: acquisition protocol, measurement definitions, independent research readings, randomized/anonymized presentation, histopathology unavailable during image review, and 4-week repeat-reading interval.
9	State whether ratings/measurements were performed independently.	Methods §§2.2-2.3: readers assessed stored examinations separately and without access to each other's measurements/classifications; image acquisition itself was not independently repeated by both readers.
10	Describe the statistical analysis of reliability/agreement.	Methods §2.6: two-way random-effects absolute-agreement single-measure ICC for interobserver reproducibility; two-way mixed-effects absolute-agreement ICC for intraobserver repeatability; Cohen's/weighted kappa, percentage agreement, Lin's CCC, and Bland-Altman analysis with 95% CIs.
11	Report the actual numbers of raters, subjects/objects, and replicate observations included in the analyses.	Results §§3.1-3.2: two readers; 320 melanomas in the final cohort; both readers performed repeat assessments after the 4-week washout.
12	Describe characteristics of the raters and study sample relevant to reliability/agreement.	Methods §§2.2-2.3 describe reader experience and review conditions; Results §3.1 and Table 1 describe the cohort, including Breslow-thickness distribution.
13	Report reliability/agreement estimates together with statistical uncertainty.	Results §§3.2-3.4 and Table 2: ICCs, kappa coefficients, Lin's CCCs, exact agreement, weighted kappa, and 95% CIs are reported.
14	Discuss the practical relevance and interpretation of reliability/agreement findings.	Discussion: findings are interpreted in relation to operator expertise, training/generalizability, measurement landmarks, distribution dependence of categorical agreement, and the complementary clinical role of UHFUS.
15	Provide detailed results in supplementary or other accessible material when useful.	Main-manuscript Table 2, the reader-specific thickness cross-tabulation tables in Results §3.4, and Figures 5-6 provide detailed reproducibility/agreement results. Supplementary Table S3 provides additional 2x2 cross-tabulations for the diagnostic-accuracy components.

Supplementary Table S3. Cross-tabulations for secondary diagnostic-accuracy analyses

S3A. 0.8-mm threshold - Reader 1

	Histopathology: Positive	Histopathology: Negative
UHFUS ≥0.8 mm: Positive	203	9
UHFUS ≥0.8 mm: Negative	8	100

S3B. 0.8-mm threshold - Reader 2

	Histopathology: Positive	Histopathology: Negative
UHFUS ≥0.8 mm: Positive	204	8
UHFUS ≥0.8 mm: Negative	7	101

S3C. Surface discontinuity versus histopathologic ulceration - Reader 1

	Ulceration: Positive	Ulceration: Negative
Surface discontinuity: Positive	48	9
Surface discontinuity: Negative	7	256

S3D. Surface discontinuity versus histopathologic ulceration - Reader 2

	Ulceration: Positive	Ulceration: Negative
Surface discontinuity: Positive	47	8
Surface discontinuity: Negative	8	257

S3E. Perilesional hypoechoic foci versus histopathologic microsatellitosis

	Microsatellitosis: Positive	Microsatellitosis: Negative
Perilesional foci: Positive	10	8
Perilesional foci: Negative	6	296
